# The association between non-suicidal self-injury and negative life events in children and adolescents in underdeveloped regions of south-western China

**DOI:** 10.7717/peerj.12665

**Published:** 2022-03-09

**Authors:** Fan Yang, Linling Jiang, Jing Miao, Xiufeng Xu, Hailiang Ran, Yusan Che, Die Fang, TianLan Wang, Yuanyuan Xiao, Jin Lu

**Affiliations:** 1 First Affiliated Hospital of Kunming Medical University, Psychiatric Department, Kunming, Yunnan, China; 2 Kunming Medical University, School of Public Health, Kunming, Yunnan, China; 3 Lincang Psychiatric Hospital, Lincang, Yunnan, China

**Keywords:** Non-suicidal self-injury, Adolescents, Cross-Sectional Studies, Negative life events, Repeated non-suicidal self-injury, Severity of non-suicidal self-injury, Underdeveloped regions of China

## Abstract

**Background:**

The association between negative life events and Non-suicidal self-injury (NSSI) in children and adolescents has been extensively discussed. Nevertheless, little is known about the relationship between negative life events and repetition and severity of NSSI. This survey aims to understand the association between NSSI prevalence, severity, repetition and the negative life events in children and adolescents in underdeveloped regions in south-western China.

**Methods:**

In this population-based cross-sectional study, 3,146 children and adolescents were included from underdeveloped regions of south-western China, and each of them requested to complete a self-assessment questionnaire. The Modified Version of Adolescents Self-Harm Scale (MASHS) and the Adolescent Self-rating negative Life Events Check-list were used to evaluate NSSI behaviors and negative life events, respectively. The statistical implementation of this study was carried out in the R statistical software, and the logistic regression analysis method was used to analyze the relationship between negative life events and adolescents’ non-suicidal self-injury behaviors.

**Results:**

The average age of all included participants was 13.31 years old. The lifetime prevalence of NSSI was 47.0% (95% CI [36.3–58.0%]). Gender, grade level and ASLEC was positively associated with NSSI. Further analysis revealed that, for all five dimensions of ASLEC, only interpersonal relationship factor (IRF) (OR 1.77 (95% CI [1.06–2.97])), health adaptation factor (HAF) (OR 2.08 (95% CI [1.31–3.31])) showed prominent association with NSSI. Multivariate Logistic regression models revealed that, repetitive NSSI (OR 4.54 (95% CI [3.66–5.63])) and NSSI severity (OR 9.01 (95% CI [6.11–13.29])) were positively associated with ASLEC.

**Conclusion:**

NSSI is very common among children and adolescents in underdeveloped regions of south-western China. Negative life event are positively associated with NSSI, repeated NSSI, and severe NSSI. Negative life events centered intervention measures might be effective in reducing NSSI among school children and adolescents in underdeveloped regions of south-western China.

## Introduction

Non-suicidal self-injury (NSSI) is currently defined by most researchers as the direct and deliberate destruction of one’s body tissue in the absence of any intent to die. NSSI includes both direct injuries such as skin cutting, carving, burning, severe scratching, needle sticking and interference with wound healing, as well as indirect injuries such as smoking, drinking, and disorderly eating (Herpertz, 1995; Nock, 2010). Direct self-injury behavior (D-SIB) is especially crippling and lethal, which is more harmful to the young. The most common form of NSSI is skin cutting, which usually occurs after a surge of negative emotions., which is transient and explosive. This behavior is considered by many researchers, as a method of relieving painful emotions and reducing tension (Nock & Prinstein, 2005; Rodham, Hawton & Evans, 2004) or as a maladaptive coping mechanism or a strategy to adjust the emotions (Favazza, 1989). In addition to being a clinically significant phenomenon in its own right, NSSI is a strong predictor of suicidal behavior (Asarnow et al., 2011; Brent, 2011; Klonsky, May & Glenn, 2013; Portzky & van Heeringen, 2007; Wilkinson et al., 2011; Wolff et al., 2013). History of NSSI is often found among those who committed or attempted suicide (Derouin & Bravender, 2004; Hawton et al., 1993; Hawton, Houston & Shepperd, 1999; Whitlock, Eckenrode & Silverman, 2006). NSSI usually starts in early adolescence (12–14 years) (Favazza, 1989; Jacobson & Gould, 2007), and peaks in later adolescence and young adulthood (Shyamala, Dianne & Keren, 2016; Skegg, 2005; Skegg et al., 2003). Remarkably high rates among adolescents and young adults make them a high-risk group (Barrocas et al., 2012; Briere & Gil, 1998; Nock & Prinstein, 2005). In recent years, NSSI has received increasing recognition as an important public health concern due to more and more NSSI behaviors in children and adolescents.

Since 2000, research on NSSI has gradually increased, but the field of NSSI research has mainly conducted in Europe and North America (such as the United States, the United Kingdom and Australia) (Gholamrezaei, De Stefano & Heath, 2017 and largely focused on Caucasian samples (Taliaferro & Muehlenkamp, 2015). Obviously, non-Western cultures and racial/ethnic minorities are underrepresented in the NSSI literature. After 2010, China’s domestic research on NSSI began to emerge, but most of them were concentrated in coastal cities or developed regions and the number of them were very limited (Pan et al., 2016). Due to the discrepancies in the assessment methodologies (sampling, tools, and time frames) and/or the different classification systems for NSSI and/or cultural differences, the results of these surveys between international and domestic regions vary greatly, which makes the conclusions of NSSI comparability poor. For example, a meta-analysis of a systematic review of 66 studies on suicidal behavior, D-SIB and NSSI in children and adolescents between 1989 and 2018 found that the lowest lifetime prevalence was 2.3% and the highest 72.2% (Lim et al., 2019). In China, a meta-analysis on NSSI among college students indicated that the prevalence rates of NSSI in the eastern, central and western regions of mainland China were 21.9, 23.0, and 2.1%, respectively (Pan et al., 2016).

The related factors of children and adolescents NSSI are complex, and it are associated with a number of risk factors such as early traumatic experiences, individual susceptibility (Yates, Carlson & Egeland, 2008), and negative life events. Negative life events refer to events that an individual feels unpleasant. These events mostly come from changes and stimuli in the individuals’ family, work, and/or learning environment, which may cause negative psychological and physiological outcomes, and cause great damage to physical and mental health (Liu et al., 1997). Even though some researchers point out that negative life events are an important factor in the survey of NSSI, but few have clarified the relationship between NSSI in children and adolescents with negative life events. Most of the research only focuses on depression, anxiety, and suicide. At the same time, in China, not only are there few studies on negative life events and NSSI in children and adolescents, but small-scale samples, or the research samples are hospitalized patients, few community samples, or the age span of the research objects were narrow, such as junior high school students in early adolescence (Shen et al., 2019; Wang, 2011; Xu, 2011) or college students in late adolescents (Li et al., 2018; Zhang et al., 2018), makes it difficult to extend the survey sample to the entire puberty. Moreover, the cultural differences and economic levels between the east and west of China are huge, resulting in a serious lack of geographical representation in existing research. Therefore, the relationship between NSSI with negative life events needs to be further studied.

At present, there are no clear guidelines for the prevention and treatment of NSSI in children and adolescents (Nock, 2012). It is particularly important to dig out relevant factors, predict high-risk groups, and formulate and implement prevention and treatment guidelines as soon as possible. There are few studies on the relationship between NSSI and negative life events in children and adolescents in remote and underdeveloped regions in south-western China, making this survey particularly important. In this survey, we aimed to understand the association between NSSI prevalence, severity, repetition and negative life events in children and adolescents in underdeveloped regions in south-western China. This will expand the overall NSSI in adolescents in communities in underdeveloped regions in western China, and provide more theoretical basis for the identification, prevention and intervention of adolescent NSSI.

## Materials and methods

### Study design

In order to understand the prevalence and relevant factors of mental health problems among school-age children and adolescents in Lincang City, we conducted a large-scale survey in Lincang County, Yunnan Province, China from December 1 to December 13, 2019. In order to reflect the scientific rationality of this survey, we used a cross-sectional survey method and a three-stage random cluster sampling method. The sampling is divided into three steps: first, randomly select from the eight districts and counties under the jurisdiction of Lincang City to Linxiang District; then, randomly select five elementary schools, five junior high schools and four high schools; finally, according to the sample size we calculated, three-four classes were randomly selected from the above-mentioned schools, and then students in the selected classes were included as subjects according to the inclusion and exclusion criteria we set up.

We have established the inclusion criteria and exclusion criteria: it must be emphasized that children who are too young often cannot understand exactly the meaning of self-harm and suicide (Mishara, 1999), but we clearly involved this content in our investigation, so we set the lower age limit above 10 years old. Inclusion criteria: (1) aged between 10 and 18 years; (2) subjects need to live in the survey area for at least 6 months per year. Exclusion criteria: (1) illiterate; (2) severe mental illness; (3) severe physical impairment; (4) communication difficulties; (5) refusal to participate. Finally, we selected a total of 3,146 qualified participants from the 14 schools identified for this survey. The Ethics Review Committee of Lincang Psychiatric Hospital (The Third People’s Hospital of Lincang) approved this study (Approval number: 2019-01), and we strictly followed the principles of the Helsinki Declaration. The participants and their legal guardians were all familiar with and signed an informed consent form prior to the investigation. Specific information such as study design, calculation of sample size and participants information can be found in our published articles (Ran et al., 2020).

### Measurements

We used a self-administered questionnaire to collect the required information. It took about 40 min for the subjects to complete the entire paper version of the self-assessment questionnaire. The contents of the self-assessment questionnaire mainly include: (1) general characteristics, (2) NSSI behavior and influence degree, (3) negative life events of adolescents, (4) psychological resilience, (5) school bullying, (6) childhood abuse; (7) suicidal ideation and behavior, *etc*. In this article, we mainly analyze the data based on the first three parts. In order to improve the quality of the self-administered questionnaires, we have pre-trained quality control personnel, who were undergraduates and postgraduates from Kunming Medical University. Each finished self-administered questionnaire was reviewed immediately by the on-site quality control personnel to reduce missed data and improve data integrity.

NSSI. The “Modified version of Adolescents Self-Harm Scale (MASHS)” questionnaire was used to collect NSSI behaviors. This questionnaire was compiled by Zheng and revised by Feng (Feng, 2008). The internal consistency reliability of the scale is 0.85, and it has satisfactory discriminative validity, standard validity and convergent validity. It is very widely-used in related studies in China. This questionnaire mainly investigates the way and severity of self-harming behaviors. The frequency of self-harming behaviors is evaluated in four levels: “0 times”, “1 times”, “2–4 times”, “5 times or more” and scored as 0, 1, 2, and 3 points; the degree of physical injury assessment points total of five levels: “none,” “mild,” “moderate,” “severe,” “very severe”, respectively, scored 0, 1, 2, 3, 4 points. Information about “Modified version of Adolescents Self-Harm Scale (MASHS)” is available in Table 1 of the Supplementary Materials.

**Table 1:** Demographic and socioeconomic information of the study sample (*N* = 3,146).

Features	Total (*N* = 3,146)	With NSSI (*N* = 1,480)	Without NSSI (*N* = 1,666)	Statistics	*P* value
Demographic					
Sex: N (%)				34.91	<0.01^a^
Boy	1,437 (45.7)	613 (41.4)	824 (49.5)		
Girl	1,709 (54.3)	867 (58.6)	842 (50.5)		
Age: Mean (SD)	13.31 (2.18)	13.89 (2.05)	12.80 (2.16)	4.72	<0.01^b^
Ethnicity: N (%)				0.05	0.82^a^
Han	2,113 (67.2)	989 (67.2)	1,124 (67.5)		
other	1,033 (32.8)	491 (32.8)	542 (32.5)		
Grade: N (%)				13.05	<0.01^a^
Primary school	1,132 (36.0)	351 (23.7)	781 (46.9)		
Junior high school	1,069 (34.0)	553 (37.4)	516 (31.0)		
Senior high school	945 (30.0)	576 (38.9)	369 (22.1)		
Socioeconomic					
Father’s education level: N (%)				2.33	0.16^a^
Elementary school and below	885 (28.1)	453 (30.6)	432 (25.9)		
Junior high school and above	1,932 (61.4)	873 (59.0)	1,059 (63.6)		
Missing and Unkonws	329 (10.5)	154 (10.4)	175 (10.5)		
Mother’s education level: N (%)				7.78	0.02^a^
Elementary school and below	1,077 (34.2)	571 (38.6)	506 (30.4)		
Junior high school and above	1,816 (57.7)	804 (54.3)	1,012 (60.7)		
Missing and Unkonws	253 (8.1)	105 (7.1)	148 (8.9)		
Only child: N (%)				4.79	0.05^a^
Yes	936 (29.8)	402 (27.2)	534 (32.1)		
No	2,210 (70.2)	1,078 (72.8)	1,132 (67.9)		
Adolescent self-rating life events: Median (IQR)					
Combined score	37 (14)	42 (15)	33 (10)	23.70	<0.01^c^
IRF (Dimension 1)	1.50 (1.00)	1.75 (1.00)	1.25 (0.50)	19.55	<0.01^c^
LPF (Dimension 2)	1.75 (1.00)	2.00 (1.25)	1.50 (1.00)	13.80	<0.01^c^
PF (Dimension 3)	1.14 (0.43)	1.29 (0.57)	1.00 (0.29)	16.55	<0.01^c^
LF (Dimension 4)	1.17 (0.50)	1.33 (0.67)	1.00 (0.33)	14.98	<0.01^c^
HAF (Dimension 5)	1.20 (0.80)	1.60 (0.80)	1.20 (0.40)	24.23	<0.01^c^
ASLEC: N (%)				282.31	<0.01^a^
Low (ASLEC ≤ 37)	1,643 (52.2)	464 (31.4)	1,179 (70.8)		
High (ASLEC > 37)	1,503 (47.8)	1016 (68.6)	487 (29.2)		

**Notes:**

ASLEC, Adolescent Self-rating Life Events Check-list; NSSI, self-harm; IRF, interpersonal relationship factor; LPF, learning pressure factor; PF, punishment factor; LF, loss factor; HAF, health adaptation factor.

a Design-based Chi-squared test.

b Design-based t-test.

c Design-based rank-sum test.

Adolescent self-rating negative life events. The Adolescent Self-rating Negative Life Events Check-list (ASLEC) is measured by using the adolescent life event scale developed by Liu et al. (1997). In 2015, Xin Xiuhong and Yao Shuqiao re-evaluated the validity and reliability of this scale and updated the norm (Xin & Yao, 2015). ASLEC has good reliability and validity, and it has better validity for adolescents, and strong pertinence, especially for middle school students. ASLEC consists of 27 items, to assess the impact of negative life events that occurred in the last 12 months, 9 months, 6 months or 3 months on the subjects occurred, if not, just mark “√” in the non-occurrence column. If it happened, it will be rated in five levels according to the psychological feeling at the time of the incident: no effect (one point), mild (two point), moderate (three point), severe (four point) or extremely severe (five point). The statistical indicators include the frequency of occurrence of the event and the amount of stress. If the event does not occur, it is counted as no effect. The cumulative score of each event is the total amount of stress. If further analysis can be divided into five factors for statistics: interpersonal relationship factor (IRF), learning pressure factor (LPF), punishment factor (PF), loss factor (LF), health adaptation factor (HAF). The total stress score for ASLEC ranges between 27 and 135, with a higher score indicates more negative life events and more stress in general. The Cronbach’s α of ASLEC in this sample was 0.849, the split-half reliability coefficient is 0.881, and the retest correlation coefficient after one week is 0.686. The last 12 months events were evaluated in this survey.

### Statistical analysis

Before conducting statistical analysis, we processed the data. In the “Modified version of Adolescents Self-Harm Scale (MASHS)” scale, according to its usage principles, we divided NSSI behaviors into two groups: (1) none (never had NSSI behaviors in the past), (2) yes (at least one NSSI behavior occurred in the past); divided into two groups according to the degree of NSSI influence: (1) mild and below (unobservable injury, mild injury: observable but not requiring treatment), (2) moderate and above (moderate injury: requires simple treatment but no need to go to a medical institution, serious injury: requires treatment in a medical institution but does not require hospitalization, very serious injury: requires emergency treatment in the emergency room and subsequent hospitalization); divided into two groups according to the frequency of NSSI: (1) non-repetitive self-injury group (the number of previous self-injuries is at most once), (2) repetitive self-injury group (the number of previous self-injuries is at least two).

The participant general characteristics are described by descriptive statistics. Frequency and rate are used to describe categorical variables, and standard deviations (SDs) and mean are used to analyze continuous variables. A univariate logistic regression model was used to screen out the possible related factors of “whether NSSI occurred”, “repetitive NSSI”, and “NSSI severity”. At this time, the significance level was set to two-tailed *P* < 0.1. Subsequently, a multivariate logistic regression model was used to calculate the corrected association between the identified factors and “whether NSSI occurred” or “repetitive NSSI” or “NSSI severity”. At this time, the significance level was set to two-tailed *P* < 0.05. All data analysis was performed in R software (R Core Team, 2021). Considering that we are using a cluster sampling design, there may be correlations between participants. Therefore, we used a related survey package, named “survey” (Lumley, 2004) and “lavaan.survey” (Oberski, 2014), to control for clustering effect throughout.

## Results

### General characteristics of the study participants

After sampling, screening, 3,234 qualified participants were initially identified. After data sorting and clearing, 88 were further excluded due to age < 10 years and age ≥ 18 years. Finally, 3,146 subjects were left for the valid analytical sample and the response rate was 97.3%. Table 1 showed the characteristics of all 3,146 participants. The average age of all included participants was 13.31 years old, with a standard deviation (SD) of 2.18. Over half (54.3%) of the participants were girls. About 30% study subjects were only child in the family. Middle school students account for a large proportion (*N* = 2014, 64%). According to the median of the ASLEC score, we divided the subjects into a low stress group (ASLEC ≤ 37) and a high stress group (ASLEC > 37). As to stress group, the high stress group accounted for 47.8%.

A total of 1,480 participants reported at least one occurrence of NSSI in their lifetime, accounting for 47.0% (95% CI [36.3–58.0%]). In NSSI adolescents, the ASLEC high stress group (68.6%) was much more common than low stress group (31.4%). Compared with “without NSSI”, “with NSSI” reported higher scores in all subtypes of ASLEC. As to gender and grade level, NSSI difference were in general obvious: girls and middle school students were observed significantly higher prevalence in NSSI behavior.

### The occurrence of NSSI

In Table 2, based on “whether NSSI behavior occurs”, univariate and multivariate logistic regression models are used respectively. First, the figure shows that the relevant variables derived from the single factor analysis with P < 0.1 are as follows: gender, grade, only child, ASLEC, mother’s education level; then, after applying multivariate adjustments, there is still significant statistical significance between “being self-harmed” and gender, grade and ASLEC. Compared to adolescents with lower stress level (ASLEC ≤ 37), those with a higher stress level (ASLEC ≤ 37) were more likely to report NSSI behaviors (OR = 4.71, 95% CI [3.49–6.36]). In order to further analyze the relationship between ASLEC and NSSI, we also included the five specific dimensions of ASLEC into the multiple logistic regression model. The results show that in these five specific dimensions, “interpersonal relationship factor” and “health adaptation factor” show a significant positive correlation, and “health adaptation factor” shows the strongest correlation: each increase in the average score by one point, the risk of NSSI increases by 2.08 times (95% CI [1.31–3.31]).

**Table 2:** Univariate and multivariable logistic regression model for NSSI.

Covariates	Univariate	Mutilvariable1	Mutilvariable2
	OR (90% CI)	OR (95% CI)	OR (95% CI)
Sex: Girls (Ref: Boys)	1.38 [1.25–1.53]	1.31 [1.11–1.54]	1.33 [1.03–1.72]
Ethnicity: Han (Ref: Other)	0.97 [0.77–1.23]		
Grade (Ref: Primary school)			
Junior high school	2.38 [1.36–4.18]	1.97 [0.95–4.10]	1.91 [0.58–6.25]
Senior high school	3.47 [2.26–5.33]	2.34 [1.39–3.94]	2.15 [0.91–5.04]
Only child: Yes (Ref: No)	0.79 [0.65–0.96]	1.00 [0.71–1.40]	
Father’s education level (Ref: Elementary and below)			
Junior high school above	0.79 [0.59–1.05]		
Mother’s education level (Ref: Elementary and below)			
Junior high school above	0.70 [0.56–0.89]	0.86 [0.69–1.06]	
ASLEC dimensions			
IRF (+1 average score)			1.77 [1.06–2.97]
LPF (+1 average score)			1.31 [1.00–1.73]
PF (+1 average score)			1.24 [0.58–2.65]
LP (+1 average score)			1.28 [0.84–1.96]
HAF (+1 average score)			2.08 [1.31–3.31]
ASLEC (Ref: Low (ASLEC ≤ 37))			
High (ASLEC > 37)	5.30 [4.40–6.39]	4.71 [3.49–6.36]	

**Note:**

ASLEC, Adolescent Self-rating Life Events Check-list.

### ASLEC with NSSI repetition and severity

We further explored the potential impact of ASLEC on repetitive NSSI and NSSI severity adolescents. The results show that “interpersonal relationship factor”, “learning stress factor”, “health adaptation factor” are positively correlated with “repetitive NSSI”, the average score of these three dimensions increases by one point, the risk of “repetitive NSSI” is respectively Increased 1.72 times (95% CI [1.20–2.45]), 1.50 times (95% CI [1.23–1.83]), 1.86 times (95% CI [1.20–2.89]). “Grades” also show correlation, compared with Primary school students, Senior high school students have a higher risk of “repetitive NSSI” (OR = 2.56, 95% CI [1.20–5.50]) (Table 3). Using the same analysis method to analyze the related factors of "NSSI severity", the following conclusions are drawn: only “health adaptation factors” is positively correlated with “NSSI severity”, the average score of these dimensions increased by one point, the risk of "NSSI severity" is respectively Increased 2.19 times (95% CI [1.47–3.24]) (Table 4).

**Table 3:** Univariate and multivariable logistic regression model for repetitive NSSI.

Covariates	Univariate	Mutilvariable1	Mutilvariable2
	OR (90% CI)	OR (95% CI)	OR (95% CI)
Sex: Girls (Ref: Boys)	1.18 [1.02–1.37]	1.10 [0.79–1.51]	1.04 [0.65–1.68]
Ethnicity: Han (Ref: Other)	0.89 [0.71–1.12]		
Grade (Ref: Primary school)			
Junior high school	2.70 [1.72–4.23]	2.24 [1.41–3.57]	2.18 [0.95–5.01]
Senior high school	4.06 [2.72–6.06]	2.74 [1.72–4.37]	2.56 [1.20–5.50]
Only child: Yes (Ref: No)	1.01 [0.84–1.22]		
Father’s education level (Ref: Elementary and below)			
Junior high school above	0.94 [0.75–1.17]		
Mother’s education level (Ref: Elementary and below)			
Junior high school above	0.78 [0.62–0.97]	0.91 [0.78–1.06]	
ASLEC dimensions			
IRF (+ 1 average score)			1.72 [1.20–2.45]
LPF (+ 1 average score)			1.50 [1.23–1.83]
PF (+ 1 average score)			1.05 [0.63–1.76]
LF (+ 1 average score)			1.12 [0.75–1.68]
HAP (+ 1 average score)			1.86 [1.20–2.89]
ASLEC (Ref: Low (ASLEC ≤ 37))			
High(ASLEC > 37)	5.44 [4.58–6.46]	4.54 [3.66–5.63]	

**Table 4:** Univariate and multivariable logistic regression model for NSSI severity.

Covariates	Univariate	Mutilvariable1	Mutilvariable2
	OR (90% CI)	OR (95% CI)	OR (95% CI)
Sex: Girls (Ref: Boys)	1.24 [1.05–1.45]	1.18 [0.95–1.48]	1.18 [0.79–1.77]
Ethnicity: Han (Ref: Other)	1.08 [0.84–1.37]		
Grade (Ref: Primary school)			
Junior high school	2.70 [1.66–4.39]	2.12 [1.31–3.43]	2.07 [0.82–5.22]
Senior high school	2.08 [1.38–3.13]	1.24 [0.83–1.83]	1.10 [0.64–1.87]
Only child: Yes (Ref: No)	0.94 [0.67–1.31]		
Father’s education level (Ref: Elementary and below)			
Junior high school above	0.98 [0.68–1.41]		
Mother’s education level (Ref: Elementary and below)			
Junior high school above	0.97 [0.68–1.38]		
ASLEC dimensions			
IRF (+1 average score)			1.69 [0.75–3.83]
LPF (+1 average score)			1.36 [0.87–2.11]
PF (+1 average score)			1.39 [0.85–2.28]
LF (+1 average score)			1.08 [0.64–1.82]
HAP (+1 average score)			2.19 [1.47–3.24]
ASLEC (Ref: Low (ASLEC ≤ 37))			
High (ASLEC > 37)	9.26 [7.10–12.08]	9.01 [6.11–13.29]	

## Discussion

We conducted a large-scale cross-sectional survey in underdeveloped regions in south-western China, investigated the epidemiology of NSSI, and explored the association between NSSI prevalence, severity, repetition and negative life events in children and adolescents. The results showed that 47% of the samples participated in NSSI, and there is evidence that NSSI is related to interpersonal relationships factor and health adaptation factor to negative life events, even after adjusting for gender, grade level, whether only child, mother’s education level.

### Epidemiological characteristics of NSSI in children and adolescents

The survey found that the detection rate of NSSI was 47%, which was very similar to the conclusion (45.6%) of Feng’s study on the incidence of NSSI using the same scale (Feng, 2008), but higher than other similar research results in China. For example, a survey of 2,200 middle school students in Shenzhen showed that the detection rate of NSSI behavior was 10.9% (Cao et al., 2019), while another meta-study analysis showed that the overall detection rate of NSSI among middle school students in Mainland China was 27.4% (95% CI [24.5–30.2%]) (Han, Xu & Su, 2017). This conclusion is also high compared with similar international studies. A previously published meta-analysis found that Global Lifetime Prevalence of Non-Suicidal Self-Injury in Children and Adolescents between 1989 and 2018 is 22.1% (95% CI [16.9–28.4%]) (Lim et al., 2019). The detection rate of NSSI behavior varies greatly among different studies, which may be due to the differences in the definition of NSSI behavior, the measurement method, the investigation tools, sample differences, the screening criteria and the cultural background. This makes the international comparability of NSSI studies decline. Therefore, it is currently necessary to develop standard investigation methods and measurement tools. When developing or quoting measurement tools, it is necessary to take full account of the local cultural background and apply them rationally and scientifically according to the cultural background in order to accurately reflect the real situation.

The results suggest sample NSSI behavior detection rate is high, and in all participants repeated self-injury rate is up to 30.4% (95% CI [21.9–41%]), moderate to severe self-injury rate is 12.1% (95% CI [8.1–18.0%]). Such a high self-harm rate and repetition rate are shocking. According to previous literature reports, self-harm and repeated NSSI are the important predictor of suicide (Gardner et al., 2019; Howe-Martin, Murrell & Guarnaccia, 2012; Klonsky, May & Glenn, 2013), and also highly predict the samples may merge other mental illnesses, such as depression, anxiety, mood disorder and personality disorder, etc. (Derouin & Bravender, 2004). Therefore, NSSI is a topic requires urgent attention and needs more in-depth discussion, especially in the underdeveloped regions of south-western China.

### Differences in demographic variables

As to NSSI, gender difference was in general obvious: girls were observed significantly higher prevalence, which was the same as the results of some foreign studies (Bresin & Schoenleber, 2015). These differences may be caused by differences in personality and mood between girls and boys. Girls in remote cities in western China are more likely to be influenced by traditional Chinese thought and have more introverted personalities. Moreover, adolescent girls are in the developmental stage, menstrual cramps and body structure changes are obvious, which makes them more sensitive, more self-esteem than boys, and are more susceptible to external factors that may cause mood swings (Tetering et al., 2020). As we all know, reducing negative emotions is the most common reason for participating in NSSI (Klonsky, 2007; Messer & Fremouw, 2008; Nock & Prinstein, 2004), which leads to more girls to use NSSI to regulate emotions more, leading to a higher detection rate of NSSI than boys. Another reason is that girls have a higher desire to be noticed and understood after NSSI than boys (McKay et al., 1996; Nam et al., 2010), which also leads to girls being more active than boys in expressing NSSI, resulting in a higher detection rate of girls. From the above results, it can be seen that in China’s children and adolescents, girls should be the high-risk group to be concerned about, and different prevention and treatment measures can be taken for different genders. Future studies can further study the various focuses of NSSI under gender differences the way.

In this study, there is a significant difference in the prevalence of NSSI among the grades of students, which is consistent with the previous research conclusions of some scholars (Zhang et al., 2016). In China, senior high school students are under the greatest learning stress and have a greater risk of bad behavior. The senior high school stage is the early stage of youth development, and their physiological and psychological development is unbalanced. As a result, when they face many sensitive issues such as sexual development, love, interpersonal relationships, and lifestyle habits, they cannot take appropriate ways to release their bad emotions (Berenbaum, Beltz & Corley, 2015). At the same time, it is easy to be impulsive at the early stage of youth development, easy to appear reverse psychology, and poor self-control ability, etc., so it is more prone to extreme NSSI. Therefore, we suggest that for senior high school students, especially senior high school girls should be the focus of attention.

### The association between NSSI prevalence, severity, repetition and negative life events

In this study, negative life events scores showed statistically significant positively correlated with NSSI prevalence, repetitive NSSI, and NSSI severity, which was consistent with previous findings (Liu et al., 2014; Tang et al., 2016; Zhang et al., 2018). NSSI is mainly related to “interpersonal relationship factor and health adaptation factor” in negative life events. The repeated NSSI behavior (NSSI frequency ≥ 2 times), after adjustment, was related to "interpersonal relationship factor, learning pressure factor and health adaptation factor. Compared with “mild NSSI”, “moderate/severe/extremely severe NSSI” after adjustment has a strong correlation with the “health adaptation factor” of negative life events. It can be found that “health adaptation factor” is all positively correlated with NSSI, repetitive NSSI and NSSI severity.

According to the construction of ASLEC, interpersonal relationships include “being misunderstood, discrimination, quarrelling with classmates and friends, and losing face in public”. Interpersonal relationships during school plays an important role in students’ cognitive, emotional, and personality development, and may even exceed family influence. Poor interpersonal relationships can lead to mental health problems in teenagers. A large number of studies have shown that NSSI behavior is positively correlated with peer isolation, bullying, alienation, bullying, etc. (Antila et al., 2017; Peng et al., 2019). Some researchers believe that NSSI behavior can alleviate the negative emotions caused by bad peer relationships, and some teenagers implement NSSI to achieve the purpose of regulating interpersonal relationships and controlling others. Some researchers studied the influence and mediating effect of “impulsive atmosphere of peer group” on NSSI among Chinese middle school students, and the results showed that the influence of the peer group has a direct effect on NSSI (You et al., 2016). In frontier cities where there are most left-behind children, most schools are boarding schools. On the one hand, the parents of students cannot give more companionship and guidance on how to get along with others. On the other hand, the boarding system allows students who are in a sensitive period to live together for a long time, resulting in a single interpersonal relationship. Once the relationship between classmates deteriorates, the entire life of the student will obviously be affected. Interpersonal relationships should be a key observation object for observing children’s mental state.

Learning pressure is a relevant factor for repetitive NSSI. The main task of children and adolescents in school is to learn, and China is under great pressure to study and is highly competitive, long-term excessive learning pressure can lead to anger, anxiety, helplessness, shame and boredom (Pekrun et al., 2002). These excessive and unnecessary emotional experiences lead to adolescents’ emotional management disorders, leading to the recurrence of NSSI behaviors (Nock, 2009). Studies have also shown that poor test scores are considered to be the two main causes of anxiety and depression in Asian children and adolescents (Kim et al., 2018). The right way for students to grow up healthily is to not only pay attention to academic performance, but to the all-round development of children.

Surprisingly, among the three models of NSSI, repetitive NSSI, and NSSI severity, “health adaptation factor” showed the strongest difference, which is different from previous research results (Tang et al., 2016; Xin & Yao, 2015). “Health adaptation factor” includes: significant changes in living habits, dislike of going to school, broken relationships, long-term distance from family and tense relationship with teachers. For further analysis, we calculated the mean value and standard deviation of each of the five questions in its dimension, and found that “dislike to go to school” had the highest score. This may be because “dislike to go to school” contains many reasons, such as poor interpersonal relationships in school, high learning pressure, unsuitable lifestyle, the attraction of other non-learning events such as electronic products. All these reasons can make students tired of learning and dislike to go to school. Therefore, "health adaptation factor" has become the most influential dimension in negative life events because of the high proportion of "don’t want to go to school".

In summary, children and adolescents who experience negative life events of a certain intensity and frequency will have a predictive and promotional effect on NSSI, repetitive NSSI, and NSSI severity, and one of the more worthy of attention is “dislike to go to school”, and it is extremely important to actively pay attention to the reasons for being tired of studying.

## Conclusions

Negative life events are an important relevant factor for children and adolescents in remote cities in south-western China, and aggravate the recurrence and severity of NSSI. Among the five special dimensions of ASLEC, “interpersonal relationship factor, learning pressure factor, and health adaptation factor” show the strongest dangerous role.

### Limitations

At the same time, this study has many limitations. First of all, this survey is a cross-sectional survey, and no causal inference can be drawn. In the future, longitudinal data can be considered to further explore the relationship between NSSI and negative life events. Second, the research object of this study is selected from remote cities in south-western China, and the participants are between 10 and 18 years old. The sample cannot fully represent the overall population of other regions. Third, we used self-reported questionnaires to collect data, which may affect the results of the survey due to personal recall bias. Finally, the present study did not explore the intermediary and regulatory factors between ASLEC and NSSI when evaluating the association between stressful negative life events and NSSI, which may lead to underestimation or miscalculation of its power. All those limitations of the present study are still the key directions for our further research.

### Future research directions

First of all, although the representativeness of the samples has been fully considered in the present study, the sample is still mainly urban middle schools. In the future, rural middle schools can be investigated to examine the differences in the content and specific performance of negative life events between the two. Secondly, with the rapid development of socio-economic and cultural of the authorities, people’s living environment has undergone great changes. Whether the negative life events that have an impact on teenagers have gradually changed by environmental changes, and what they are, remains to be further studied. In the future research, we can include some events with the characteristics of the current era according to the characteristics of the current youth’s life, such as the phenomenon of left-behind children, such as online socialization brought by electronic products, excessive use of smartphones and networks, *etc*. These are expected to become an important part of the source of negative life events.

## Supplemental Information

10.7717/peerj.12665/supp-1 Supplemental Information 1 Codebook.

10.7717/peerj.12665/supp-2 Supplemental Information 2 Raw data.

## Additional Information and Declarations

### Data Availability

The following information was supplied regarding data availability: The raw measurements are available in the Supplemental File.

## References

[ref-1] Antila H, Arola R, Hakko H, Riala K, Riipinen P, Kantojarvi L (2017). Bullying involvement in relation to personality disorders: a prospective follow-up of 508 inpatient adolescents. European Child & Adolescent Psychiatry.

[ref-2] Asarnow JR, Porta G, Spirito A, Emslie G, Clarke G, Wagner KD, Vitiello B, Keller M, Birmaher B, McCracken J, Mayes T, Berk M, Brent DA (2011). Suicide attempts and nonsuicidal self-injury in the treatment of resistant depression in adolescents: findings from the TORDIA study. Journal of the American Academy of Child & Adolescent Psychiatry.

[ref-3] Barrocas AL, Hankin BL, Young JF, Abela JR (2012). Rates of nonsuicidal self-injury in youth: age, sex, and behavioral methods in a community sample. Pediatrics.

[ref-4] Berenbaum SA, Beltz AM, Corley R (2015). The importance of puberty for adolescent development: conceptualization and measurement. Advances in Child Development and Behavior.

[ref-5] Brent D (2011). Nonsuicidal self-injury as a predictor of suicidal behavior in depressed adolescents. American Journal of Psychiatry.

[ref-6] Bresin K, Schoenleber M (2015). Gender differences in the prevalence of nonsuicidal self-injury: a meta-analysis. Clinical Psychology Review.

[ref-7] Briere J, Gil E (1998). Self-mutilation in clinical and general population samples: prevalence, correlates, and functions. American Journal of Orthopsychiatry.

[ref-8] Cao Xl, Wen SY, Liu JB, Lu JP (2019). Study on the prevalence and risk factors of non-suicidal self-injury among middle school students in Shenzhen. Sichuan Mental Health.

[ref-9] Derouin A, Bravender T (2004). Living on the edge: the current phenomenon of self-mutilation in adolescents. MCN, The American Journal of Maternal/Child Nursing.

[ref-10] Favazza AR (1989). Why patients mutilate themselves. Hospital & Community Psychiatry.

[ref-11] Feng Y (2008). The relation of adolecents’ self-harm behaviors, individual emotion characteristics and family environment factorsmaster.

[ref-12] Gardner W, Pajer K, Cloutier P, Currie L, Colman I, Zemek R, Hatcher S, Lima I, Cappelli M (2019). Health outcomes associated with emergency department visits by adolescents for self-harm: a propensity-matched cohort study. Canadian Medical Association Journal.

[ref-13] Gholamrezaei M, De Stefano J, Heath NL (2017). Nonsuicidal self-injury across cultures and ethnic and racial minorities: a review. International Journal of Psychology.

[ref-14] Han AZ, Xu G, Su PY (2017). A meta-analysis of characteristics of non-suicidal self-injury among middle school students in mainland China. Chinese Journal of School Health.

[ref-15] Hawton K, Fagg J, Platt S, Hawkins M (1993). Factors associated with suicide after parasuicide in young people. BMJ.

[ref-16] Hawton K, Houston K, Shepperd R (1999). Suicide in young people. Study of 174 cases, aged under 25 years, based on coroners’ and medical records. British Journal of Psychiatry.

[ref-17] Herpertz S (1995). Self-injurious behaviour. Psychopathological and nosological characteristics in subtypes of self-injurers. Acta Psychiatrica Scandinavica.

[ref-18] Howe-Martin LS, Murrell AR, Guarnaccia CA (2012). Repetitive nonsuicidal self-injury as experiential avoidance among a community sample of adolescents. Journal of Clinical Psychology.

[ref-19] Jacobson CM, Gould M (2007). The epidemiology and phenomenology of non-suicidal self-injurious behavior among adolescents: a critical review of the literature. Archives of Suicide Research.

[ref-20] Kim YJ, Moon SS, Lee JH, Kim JK (2018). Risk factors and mediators of suicidal ideation among Korean Adolescents. Crisis.

[ref-21] Klonsky ED (2007). The functions of deliberate self-injury: a review of the evidence. Clinical Psychology Review.

[ref-22] Klonsky ED, May AM, Glenn CR (2013). The relationship between nonsuicidal self-injury and attempted suicide: converging evidence from four samples. Journal of Abnormal Psychology.

[ref-23] Li HH, He H, Wang YY, Gu JY, Liu WJ, Mao SJ, Tang HM, Fu YY, Chen XL, Huang P (2018). Effects of negative life events on non-suicidal self-injury behaviors in college students. Journal of Nanchang University (Medical Sciences).

[ref-24] Lim KS, Wong CH, McIntyre RS, Wang J, Zhang Z, Tran BX, Tan W, Ho CS, Ho RC (2019). Global lifetime and 12-month prevalence of suicidal behavior, deliberate self-harm and non-suicidal self-injury in children and adolescents between 1989 and 2018: a meta-analysis. International Journal of Environmental Research and Public Health.

[ref-25] Liu RT, Frazier EA, Cataldo AM, Simon VA, Spirito A, Prinstein MJ (2014). Negative life events and non-suicidal self-injury in an adolescent inpatient sample. Archives of Suicide Research.

[ref-26] Liu XC, Liu LQ, Yang J, Chai FX, Wang AZ, Sun LM, Zhao GF, Ma DD (1997). Compilation and reliability and validity test of adolescent life event scale. Shandong Archires of Psychiatry.

[ref-27] Lumley T (2004). Analysis of complex survey samples. Journal of Statistical Software.

[ref-28] McKay JR, Rutherford MJ, Cacciola JS, Kabasakalian-McKay R, Alterman AI (1996). Gender differences in the relapse experiences of cocaine patients. Journal of Nervous and Mental Disease.

[ref-29] Messer JM, Fremouw WJ (2008). A critical review of explanatory models for self-mutilating behaviors in adolescents. Clinical Psychology Review.

[ref-30] Mishara BL (1999). Conceptions of death and suicide in children ages 6–12 and their implications for suicide prevention. Suicide and Life Threatening Behavior.

[ref-31] Nam SK, Chu HJ, Lee MK, Lee JH, Kim N, Lee SM (2010). A meta-analysis of gender differences in attitudes toward seeking professional psychological help. Journal of American College Health.

[ref-32] Nock MK (2009). Why do people hurt themselves? New insights into the nature and functions of self-injury. Current Directions in Psychological Science.

[ref-33] Nock MK (2010). Self-injury. Annual Review of Clinical Psychology.

[ref-34] Nock MK (2012). Future directions for the study of suicide and self-injury. Journal of Clinical Child & Adolescent Psychology.

[ref-35] Nock MK, Prinstein MJ (2004). A functional approach to the assessment of self-mutilative behavior. Journal of Consulting and Clinical Psychology.

[ref-36] Nock MK, Prinstein MJ (2005). Contextual features and behavioral functions of self-mutilation among adolescents. Journal of Abnormal Psychology.

[ref-37] Oberski D (2014). lavaan.survey: an R package for complex survey analysis of structural equation models. Journal of Statistical Software.

[ref-38] Pan Z, Mao SJ, Tang HM, Fu YY, Sun WX, Liao ZL, Li JN, Qiu HH, Zhu JY, Huang P (2016). Meta-analysis on non-suicidal self-injury among college students in China. Chinese Journal of School Health.

[ref-39] Pekrun R, Goetz T, Titz W, Perry RPJEP (2002). Academic emotions in students’ self-regulated learning and achievement: a program of qualitative and quantitative research. Educational Psychologist.

[ref-40] Peng Z, Klomek AB, Li L, Su X, Sillanmaki L, Chudal R, Sourander A (2019). Associations between Chinese adolescents subjected to traditional and cyber bullying and suicidal ideation, self-harm and suicide attempts. BMC Psychiatry.

[ref-41] Portzky G, van Heeringen K (2007). Deliberate self-harm in adolescents. Current Opinion in Psychiatry.

[ref-61] R Core Team (2021). https://www.r-project.org.

[ref-42] Ran H, Cai L, He X, Jiang L, Wang T, Yang R, Xu X, Lu J, Xiao Y (2020). Resilience mediates the association between school bullying victimization and self-harm in Chinese adolescents. Journal of Affective Disorders.

[ref-43] Rodham K, Hawton K, Evans E (2004). Reasons for deliberate self-harm: comparison of self-poisoners and self-cutters in a community sample of adolescents. Journal of the American Academy of Child & Adolescent Psychiatry.

[ref-44] Shen XS, Zhong GH, Mo DM, Cai Y, Sun Y (2019). Relationship among suicide ideation, self-injurious behavior and life events of junior high school students. China Journal of Health Psychology.

[ref-45] Shyamala NR, Dianne M, Keren S (2016). A population-based study of help-seeking for self-harm in young adults. Australian & New Zealand Journal of Psychiatry.

[ref-46] Skegg K (2005). Self-harm. Lancet.

[ref-47] Skegg K, Nada-Raja S, Dickson N, Paul C, Williams S (2003). Sexual orientation and self-harm in men and women. American Journal of Psychiatry.

[ref-48] Taliaferro LA, Muehlenkamp JJ (2015). Risk factors associated with self-injurious behavior among a national sample of undergraduate college students. Journal of American College Health.

[ref-49] Tang J, Yang W, Ahmed NI, Ma Y, Liu HY, Wang JJ, Wang PX, Du YK, Yu YZ (2016). Stressful life events as a predictor for nonsuicidal self-injury in Southern Chinese adolescence: a cross-sectional study. Medicine.

[ref-50] Tetering M, Laan AMV, Kogel CH, Groot RHM, Jolles J (2020). Sex differences in self-regulation in early, middle and late adolescence: a large-scale cross-sectional study. PLOS ONE.

[ref-51] Wang L (2011). The epidemiological survey and correlation analysis about delibrate self-harm behavior of junior school students in Dalian.

[ref-52] Whitlock J, Eckenrode J, Silverman D (2006). Self-injurious behaviors in a college population. Pediatrics.

[ref-53] Wilkinson P, Kelvin R, Roberts C, Dubicka B, Goodyer I (2011). Clinical and psychosocial predictors of suicide attempts and nonsuicidal self-injury in the adolescent depression antidepressants and psychotherapy trial (ADAPT). American Journal of Psychiatry.

[ref-54] Wolff J, Frazier EA, Esposito-Smythers C, Burke T, Sloan E, Spirito A (2013). Cognitive and social factors associated with NSSI and suicide attempts in psychiatrically hospitalized adolescents. Journal of Abnormal Child Psychology.

[ref-55] Xin XH, Yao SQ (2015). Validity and reliability of the adolescent self-rating life events checklist in middle school students. Chinese Mental Health Journal.

[ref-56] Xu ZW (2011). The self-injurious behavior and its influencing factors among secondary school students in the countryside of Anhui Province.

[ref-57] Yates TM, Carlson EA, Egeland B (2008). A prospective study of child maltreatment and self-injurious behavior in a community sample. Development and Psychopathology.

[ref-58] You J, Zheng C, Lin MP, Leung F (2016). Peer group impulsivity moderated the individual-level relationship between depressive symptoms and adolescent nonsuicidal self-injury. Journal of Adolescence.

[ref-59] Zhang SC, Tao FB, Wu XY, Tao SM, Fang J (2016). Low health literacy and psychological symptoms potentially increase the risks of non-suicidal self-injury in Chinese middle school students. BMC Psychiatry.

[ref-60] Zhang Y, Wang JF, Wang YQ, Chen ZP, Duan Y, Wang J, Jin QL, Yao YS (2018). The influence of gender and life events of adolescents on the self-injury behavior of college students. Journal of Qiqihar Medical College.

