# Supplementary material for: The association between non-suicidal self-injury and negative life events in children and adolescents in underdeveloped regions of south-western China
Source: PeerJ. 2022 Mar 9;10:e12665. doi: 10.7717/peerj.12665 (PMC8917796; doi:10.7717/peerj.12665)
Supplement: Supplemental Information 1 [file peerj-10-12665-s001.docx]

| **A1 Gender** | | |
| --- | --- | --- |
| 1 | | Boy |
| 2 | | Girl |
| **A2 Ethnic** | | |
| 1 | | Han |
| 2 | | Bai |
| 3 | | Yi |
| 4 | | Hui |
| 5 | | Wa |
| 6 | | Other |
| **A3 Date of birth** | | |
|  | | |
| **A7 Grade** | | |
| 1 | | Primary school |
| 2 | | Junior high school |
| 3 | | Senior high school |
|  | | |
| **A8 Study style** | |  |
| 1 | | Day students |
| 2 | | Boarding students |
| **A9 If an only child** | | |
| 1 | | Yes |
| 2 | | No |
| **A10 Number of school transfers since elementary school (excluding school changes due to graduation)** | | |
|  |  | |
| **A111 How many people you are currently living with on a long-term basis (excluding yourself, living with you for at least 6 months of the year)** | | |
|  | | |
| **A1121 Live with Mother** | | |
| 1 | | Yes |
| 2 | | No |
| **A1122 Live with father** | | |
| 1 | | Yes |
| 2 | | No |
| **A1123 Live with grandparents** | | |
| 1 | | Yes |
| 2 | | No |
| **A1124 Live with brothers and sisters** | | |
| 1 | | Yes |
| 2 | | No |
| **A1125 Live with cousins** | | |
| 1 | | Yes |
| 2 | | No |
| **A1126 Live with father's brothers or sisters** | | |
| 1 | | Yes |
| 2 | | No |
| **A1127 Live with mother's brothers or sisters** | | |
| 1 | | Yes |
| 2 | | No |
| **A1128 Live with others** | | |
| 1 | | Yes |
| 2 | | No |
| **A121 Is your father alive？** | | |
| 1 | | Yes |
| 2 | | No |
| **A1221 If you know your father’s age?** | | |
| 1 | | Yes |
| 2 | | No |
| **A1222 Your father's exact age?** | | |
|  | | |
| **A123 Father's education level** | | |
| 0 | | Unknown |
| 1 | | Illiteracy and below |
| 2 | | Elementary school |
| 3 | | Junior high school |
| 4 | | Senior high school |
| 5 | | College and above |
| **A1241 Physical disability** | |  |
| 1 | | Yes |
| 2 | | No |
| **A1242 Mental illness** | | |
| 1 | | Yes |
| 2 | | No |
| **A1243 Hypertension** | | |
| 1 | | Yes |
| 2 | | No |
| **A1244 Diabetes** | | |
| 1 | | Yes |
| 2 | | No |
| **A1245 Chronic hepatitis** | | |
| 1 | | Yes |
| 2 | | No |
| **A1246 Tuberculosis** | | |
| 1 | | Yes |
| 2 | | No |
| **A1247 Malignant tumor** | | |
| 1 | | Yes |
| 2 | | No |
| **A1248 Coronary heart disease** | | |
| 1 | | Yes |
| 2 | | No |
| **A1249 Pneumoconiosis/silicosis** | | |
| 1 | | Yes |
| 2 | | No |
| **A12410 Arthritis** | | |
| 1 | | Yes |
| 2 | | No |
| **A12411 Cerebrovascular disease and sequelae** | | |
| 1 | | Yes |
| 2 | | No |
| **A12412 Cataract** | | |
| 1 | | Yes |
| 2 | | No |
| **A12413 Other** | | |
| 1 | | Yes |
| 2 | | No |
| **A12415 No disease** | | |
| 1 | Yes | |
| 2 | No | |
| **A1211 How old were you when your father died?** | | |
|  | | |
| **A125 Is your mother alive？** | | |
| 1 | | Yes |
| 2 | | No |
| **A1261 If you know your mother’s age?** | | |
| 1 | | Yes |
| 2 | | No |
| **A1262 Your mother's exact age?** | | |
|  | | |
| **A127 Mother's education level** | | |
| 0 | | Unknown |
| 1 | | Illiteracy and below |
| 2 | | Elementary school |
| 3 | | Junior high school |
| 4 | | Senior high school |
| 5 | | College and above |
| **A1281 Physical disability** | |  |
| 1 | | Yes |
| 2 | | No |
| **A1282 Mental illness** | | |
| 1 | | Yes |
| 2 | | No |
| **A1283 Hypertension** | | |
| 1 | | Yes |
| 2 | | No |
| **A1284 Diabetes** | | |
| 1 | | Yes |
| 2 | | No |
| **A1285 Chronic hepatitis** | | |
| 1 | | Yes |
| 2 | | No |
| **A1286 Tuberculosis** | | |
| 1 | | Yes |
| 2 | | No |
| **A1287 Malignant tumor** | | |
| 1 | | Yes |
| 2 | | No |
| **A1288 Coronary heart disease** | | |
| 1 | | Yes |
| 2 | | No |
| **A1289 Pneumoconiosis/silicosis** | | |
| 1 | | Yes |
| 2 | | No |
| **A12810 Arthritis** | | |
| 1 | | Yes |
| 2 | | No |
| **A12811 Cerebrovascular disease and sequelae** | | |
| 1 | | Yes |
| 2 | | No |
| **A12812 Cataract** | | |
| 1 | | Yes |
| 2 | | No |
| **A12813 Other** | | |
| 1 | | Yes |
| 2 | | No |
| **A12815 No disease** | | |
| 1 | | Yes |
| 2 | | No |
| **A1251 How old were you when mom died** | | |
|  | |  |
| **A132 In the past** **one year, whether your father accumulates to go out to work half a year above?** | | |
| 1 | | Yes |
| 2 | | No |
| **A139 In the past one year, whether your mother has been out working for more than half a year?** | | |
| 1 | | Yes |
| 2 | | No |
| **E11 Whether the occurrence times of NSSI are all zero** | | |
| 1 | | Yes |
| 2 | | No |
| **E12** **Times of intentionally cutting your skin with glass or a knife** | | |
| 1 | zero | |
| 2 | 1 time | |
| 3 | 2-4 times | |
| 4 | At least 5 times | |
| **E13 The degree of** **damage** | | |
| 1 | No | |
| 2 | Mild | |
| 3 | Moderate | |
| 4 | Severe | |
| 5 | Extremely Severe | |
| **E21** **Times of deliberately poking open the wound to prevent the wound from healing****(The classification method is the same as E12)** | | |
| **E22 The degree of damage****(The classification method is the same as E13)** | | |
| **E31 Times of deliberately burn/scald your skin with cigarette butts, lighters or other things(The classification method is the same as E12)** | | |
| **E32 The degree of damage(The classification method is the same as E13)** | | |
| **E41 Times of Deliberately tattooing or patterning on the body (except for tattooing)(The classification method is the same as E12)** | | |
| **E42 The degree of damage(The classification method is the same as E13)** | | |
| **E51 Times of deliberately scrape and bleed your skin(The classification method is the same as E12)** | | |
| **E52 The degree of damage(The classification method is the same as E13)** | | |
| **E61 Times of Deliberately pierce something into the skin or under the nail (The classification method is the same as E12)** | | |
| **E62 The degree of damage(The classification method is the same as E13)** | | |
| **E71 Times of Intentionally hit something with the head, causing bruises (The classification method is the same as E12)** | | |
| **E72 The degree of damage(The classification method is the same as E13)** | | |
| **E81 Times of deliberately plucking your own hair (The classification method is the same as E12)** | | |
| **E82 The degree of damage(The classification method is the same as E13)** | | |
| **E91 Times of deliberately hitting hard objects such as walls or glass with your hands (The classification method is the same as E12)** | | |
| **E92 The degree of damage(The classification method is the same as E13)** | | |
| **E101 Times of deliberately scratching yourself violently, reaching the level of scars or bleeding (The classification method is the same as E12)** | | |
| **E102 The degree of damage(The classification method is the same as E13)** | | |
| **E111 Times of deliberately puncturing a certain part of the body with a needle, nail or other things to bleed (The classification method is the same as E12)** | | |
| **E112 The degree of damage(The classification method is the same as E13)** | | |
| **E121 Times of deliberately rubbing the skin to bleed (The classification method is the same as E12)** | | |
| **E122 The degree of damage(The classification method is the same as E13)** | | |
| **E131 Times of beat yourself deliberately, causing bruises (The classification method is the same as E12)** | | |
| **E132 The degree of damage(The classification method is the same as E13)** | | |
| **E141 Times of deliberately use a rope or other things to strangle your wrists and other parts (The classification method is the same as E12)** | | |
| **E142 The degree of damage(The classification method is the same as E13)** | | |
| **E151 Times of intentionally let others hit or bite yourself in order to harm your own body (The classification method is the same as E12)** | | |
| **E152 The degree of damage(The classification method is the same as E13)** | | |
| **E161 Times of deliberately get yourself electrocuted without life-threatening conditions (The classification method is the same as E12)** | | |
| **E162 The degree of damage(The classification method is the same as E13)** | | |
| **E171 Times of bite yourself deliberately causing skin breakage (The classification method is the same as E12)** | | |
| **E172 The degree of damage(The classification method is the same as E13)** | | |
| **E181** **Times of deliberately ignite or touch the flame in your hand (The classification method is the same as E12)** | | |
| **E182 The degree of damage(The classification method is the same as E13)** | | |
| **P1 Misunderstood or blamed** | | |
| 1 | Yes | |
| 2 | No | |
| **P1111 The level of distress the event has caused you** | | |
| 1 | No | |
| 2 | Mild | |
| 3 | Moderate | |
| 4 | Severe | |
| 5 | Extremely Severe | |
| **P2 Discrimination or cold reception** | | |
| 1 | Yes | |
| 2 | No | |
| **P2111 The level of distress the event has caused you** | | |
| 1 | No | |
| 2 | Mild | |
| 3 | Moderate | |
| 4 | Severe | |
| 5 | Extremely Severe | |
| **P3 Exam failure or poor grades** | | |
| 1 | Yes | |
| 2 | No | |
| **P31 The level of distress the event has caused you** | | |
| 1 | No | |
| 2 | Mild | |
| 3 | Moderate | |
| 4 | Severe | |
| 5 | Extremely Severe | |
| **P4 Disputes with classmates or friends(The classification method is the same as P3)** | | |
| **P41 The level of distress the event has caused you(The classification method is the same as P31)** | | |
| **P5 Significant changes in lifestyle (eating, rest, etc.)(The classification method is the same as P3)** | | |
| **P51 The level of distress the event has caused you(The classification method is the same as P31)** | | |
| **P6 I don't like to go to school(The classification method is the same as P3)** | | |
| **P61 The level of distress the event has caused you(The classification method is the same as P31)** | | |
| **P7 Unsuccessful love or broken love(The classification method is the same as P3)** | | |
| **P71 The level of distress the event has caused you(The classification method is the same as P31)** | | |
| **P8 A Long-term away from family members cannot be reunited(The classification method is the same as above)(The classification method is the same as P3)** | | |
| **P81 The level of distress the event has caused you(The classification method is the same as P31)** | | |
| **P9 Heavy learning burden(The classification method is the same as P3)** | | |
| **P91 The level of distress the event has caused you(The classification method is the same as P31)** | | |
| **P10 Tension with a teacher(The classification method is the same as P3)** | | |
| **P101 The level of distress the event has caused you(The classification method is the same as P31)** | | |
| **P11 I am seriously ill(The classification method is the same as P3)** | | |
| **P111 The level of distress the event has caused you(The classification method is the same as P31)** | | |
| **P12 Relatives and/or friends are seriously ill(The classification method is the same as P3)** | | |
| **P121 The level of distress the event has caused you(The classification method is the same as P31)** | | |
| **P13 Death of relatives (friends)(The classification method is the same as P3)** | | |
| **P131 The level of distress the event has caused you(The classification method is the same as P31)** | | |
| **P14 Stolen or lost things(The classification method is the same as P3)** | | |
| **P141 The level of distress the event has caused you(The classification method is the same as P31)** | | |
| **P15 Lose face in public(The classification method is the same as P3)** | | |
| **P151 The level of distress the event has caused you(The classification method is the same as P31)** | | |
| **P16 Family financial difficulties(The classification method is the same as P3)** | | |
| **P161 The level of distress the event has caused you(The classification method is the same as P31)** | | |
| **P17 There are conflicts within the family(The classification method is the same as P3)** | | |
| **P171 The level of distress the event has caused you(The classification method is the same as P31)** | | |
| **P18 Expected selections (such as various evaluations) failed(The classification method is the same as P3)** | | |
| **P181 The level of distress the event has caused you(The classification method is the same as P31)** | | |
| **P19 Criticized or punished(The classification method is the same as P3)** | | |
| **P191 The level of distress the event has caused you(The classification method is the same as P31)** | | |
| **P20 Transfer or suspension(The classification method is the same as P3)** | | |
| **P201 The level of distress the event has caused you(The classification method is the same as P31)** | | |
| **P21 fined(The classification method is the same as P3)** | | |
| **P211 The level of distress the event has caused you(The classification method is the same as P31)** | | |
| **P22 Academic pressure(The classification method is the same as P3)** | | |
| **P221 The level of distress the event has caused you(The classification method is the same as P31)** | | |
| **P23 Fight with people(The classification method is the same as P3)** | | |
| **P231 The level of distress the event has caused you(The classification method is the same as P31)** | | |
| **P24 Beaten and scolded by parents(The classification method is the same as P3)** | | |
| **P241 The level of distress the event has caused you(The classification method is the same as P31)** | | |
| **P25 Family puts pressure on you to study(The classification method is the same as P3)** | | |
| **P251 The level of distress the event has caused you(The classification method is the same as P31)** | | |
| **P26 Unexpected fright or accident** **(The classification method is the same as P3)** | | |
| **P261 The level of distress the event has caused you(The classification method is the same as P31)** | | |
| **P27 Other setbacks (The classification method is the same as P3)** | | |
| **P271 The level of distress the event has caused you(The classification method is the same as P31)** | | |
